# Dopant-Dependent Toxicity of CeO_2_ Nanoparticles Is Associated with Dynamic Changes in H3K4me3 and H3K27me3 and Transcriptional Activation of NRF2 Gene in HaCaT Human Keratinocytes

**DOI:** 10.3390/ijms22063087

**Published:** 2021-03-17

**Authors:** Jang Hyun Choi, Haram Lee, Hangil Lee, Hansol Lee

**Affiliations:** 1 Department of Biological Sciences, College of Natural Science, Inha University, 100 Inha-ro, Michuhol-gu, Incheon 22212, Korea; jchoi@inha.ac.kr (J.H.C.); 22192089@inha.edu (H.L.); 2 Department of Chemistry, Sookmyung Women’s University, Seoul 04310, Korea

**Keywords:** cerium oxide nanoparticles (CeO_2_ NPs), transition metal doping, reactive oxygen species (ROS), NRF2-KEAP1 pathway, histone lysine methylation

## Abstract

Despite advances in the preparation of metal oxide (MO) nanoparticles (NPs) as catalysts for various applications, concerns about the biosafety of these particles remain. In this study, we prepared transition metal-doped cerium oxide (TM@CeO_2_; TM = Cr, Mn, Fe, Co, or Ni) nanoparticles and investigated the mechanism underlying dopant-dependent toxicity in HaCaT human keratinocytes. We show that doping with Cr or Co but not Fe, Mn, or Ni increased the toxicity of CeO_2_ NPs in dose- and time-dependent manners and led to apoptotic cell death. Interestingly, while both undoped and transition metal-doped NPs increased intracellular reactive oxygen species (ROS), toxic Cr@CeO_2_ and Co@CeO_2_ NPs failed to induce the expression of NRF2 (nuclear factor erythroid 2-related factor 2) as well as its downstream target genes involved in the antioxidant defense system. Moreover, activation of NRF2 transcription was correlated with dynamic changes in H3K4me3 and H3K27me3 at the promoter of NRF2, which was not observed in cells exposed to Cr@CeO_2_ NPs. Furthermore, exposure to relatively non-toxic Fe@CeO_2_ NPs, but not the toxic Cr@CeO_2_ NPs, resulted in increased binding of MLL1 complex, a major histone lysine methylase mediating trimethylation of histone H3 lysine 4, at the NRF2 promoter. Taken together, our findings strongly suggest that failure of cells to respond to oxidative stress is critical for dopant-dependent toxicity of CeO_2_ NPs and emphasize that careful evaluation of newly developed NPs should be preceded before industrial or biomedical applications.

## 1. Introduction

Metal oxide nanoparticles (MONPs) have been used for various chemical and biological applications, for example, as chemical sensors, biosensors, drug delivery agents, and for cancer therapy and in electrochemical reactions, due to their unique physicochemical properties [1,2,3,4]. MONPs are produced and consumed in large quantities, and the breadths of their applications are rapidly expanding. However, concerns have been expressed regarding their adverse effects on human health and the environment, as MONPs could enter the human body through ingestion, infection, inhalation, or skin contact [5,6,7,8,9]. The toxicities of MONPs depend on particle size and surface area, dosage, exposure time, pH, and extent of agglomeration [7,10,11,12,13,14]. In vitro and in vivo studies have suggested that induction of reactive oxygen species (ROS) by MONPs predominantly underlies their toxicities by causing oxidative stress and inflammation, leading to intracellular component damage and aberrant expressions of genes associated with cellular homeostasis [7,15]. In addition, changes in epigenetic modification, such as DNA methylation and histone modification, have recently been suggested as alternative mechanisms of MONPs-mediated toxicity [16]. However, the effects of MONPs on histone modification, especially at the ROS-related genes, and the effects of histone modifications on MONPs-mediated toxicity are not fully understood.

CeO_2_ is a lanthanide element metal oxide, and CeO_2_ nanoparticles (NPs) have been used in wide-ranging applications, such as photo-catalysts, solid-oxide fuel cells, and dye-sensitized solar cells [17,18,19]. CeO_2_ NPs are also being considered for potential biological and biomedical applications because of their ability to mimic the actions of enzymes, such as superoxide dismutase (SOD) and catalase [20,21,22]. In addition, various strategies (e.g., synthetic protocol, metal (anion) doping, and physicochemical property modifications) have been developed to enhance the activities of CeO_2_ NPs. In particular, transition metal doping has been proven to be effective in enhancing photocatalytic activity [23,24,25,26,27,28,29]. As has been performed for other MONPs, the toxicities of CeO_2_ NPs have been evaluated in various cellular and organismal contexts, but published results are inconclusive due, at least in part, to differences between the physicochemical properties of the CeO_2_ NPs tested and cell-type dependent responsiveness [30,31,32,33,34,35,36,37,38,39]. Although most studies have reported at best modest toxic effects or even protective effects [33,34,35], some have suggested CeO_2_ NPs may be toxic and cause cell death, presumably due to oxidative stress (e.g., reactive oxygen species (ROS) production), DNA damage, alterations in cell signaling, and deregulated gene expression [36,37,38,39].

NRF2 (nuclear factor erythroid 2-related factor 2) is a transcription factor that controls the cellular antioxidant defense system [40]. Its function is mainly regulated at the posttranscriptional level. Upon the oxidative stimuli, NRF2 is freed from KEAP1 (Kelch like ECH associated protein 1), a negative regulator of NRF2, and enters the nucleus, where it activates an array of antioxidative metabolizing/detoxifying genes by binding to ATE (antioxidant response element) [41,42]. NRF2 is also regulated at the transcriptional level. Studies have shown that transcription factors, such as AhR, NF-kB, and even NRF2 itself, regulate the expression of NRF2 [43,44,45]. In addition, epigenetic modifications, such as DNA methylation and histone methylation, have recently been reported to be key regulators of NRF2 [46].

The effects of CeO_2_ NPs on NRF2-KEAP1 signaling have been reported in several studies, but results are not conclusive [38,47,48,49,50]. It has been shown that exposure to CeO_2_ NPs induces oxidative stresses, increases nuclear NRF2 level, and eventually causes cell death [38]. However, it has also been reported CeO_2_ NPs have protective effects due to the transcriptional and posttranscriptional activation of NRF2 signaling [47,48], and yet others have reported CeO_2_ NPs exposure resulted in no significant change or even a reduction in NRF2 level [49,50]. Moreover, the effect of CeO_2_ NPs on the epigenetic modification of the *NRF2* gene has not been studied in detail. In this study, we synthesized five different TM@CeO_2_ NPs (where TM = Cr, Mn, Fe, Co, or Ni) and investigated their effects on HaCaT human keratinocytes and the mechanism responsible for dopant-dependent toxicity. Our comparative analysis provides evidence that transcriptional activation of the *NRF2* gene and dynamic changes in H3K4me3 and H3K27me3 histone modifications play a critical role in dopant-dependent toxicity of TM@CeO_2_ NPs.

## 2. Results and Discussion

### 2.1. Effects of Transition Metal Doping on Cell Viability

**Figure 1:**
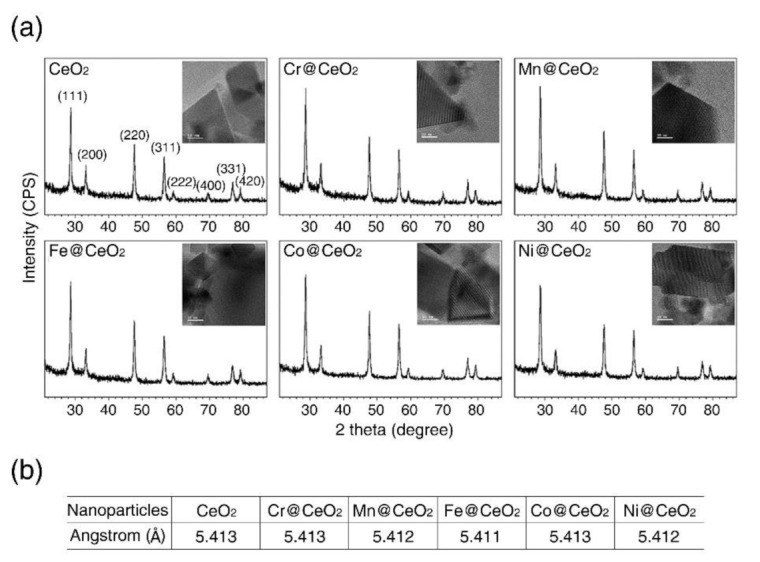
Effect of transition metal doping on the surface structure of CeO_2_ NPs. (**a**) X-ray diffraction (XRD) analysis of undoped CeO_2_ and TM@CeO_2_ NPs. The insets present corresponding transmission electron microscopy (TEM) images. (scale bar is 10 nm) (**b**) Lattice constant of CeO_2_ and TM@CeO_2_ NPs.

To investigate the effects of transition metal doping on the toxicity of CeO_2_ NPs, we first analyzed the crystal structure of TM@CeO_2_ NPs by X-ray diffraction (XRD) and transmission electron microscopy (TEM). The XRD pattern of CeO_2_ NPs was typical of fluorite structured CeO_2_ without any obvious structural changes. All tested TM@CeO_2_ NPs generated XRD spectra with peaks at 2θ = 28.7°, 33.2°, 47.7°, 56.5°, 59.2°, 69.5°, 76.9°, and 79.3° (Figure 1a), which corresponded to the reflections from the (111), (200), (220), (311), (222), (400), (331), and (420) planes of undoped CeO_2_ NPs (JCPDS card No. 41–1455). TEM images demonstrated undoped CeO_2_ and TM@CeO_2_ NPs had similar sizes (~20 nm) and shapes (Figure 1a, inset). In addition, the *c* axis lattice constants of TM@CeO_2_ NPs were almost the same as that of undoped CeO_2_ NPs (Figure 1b). These observations suggest that transition metal doping is unlikely to cause significant changes in the surface structures of CeO_2_ NPs.

**Figure 2:**
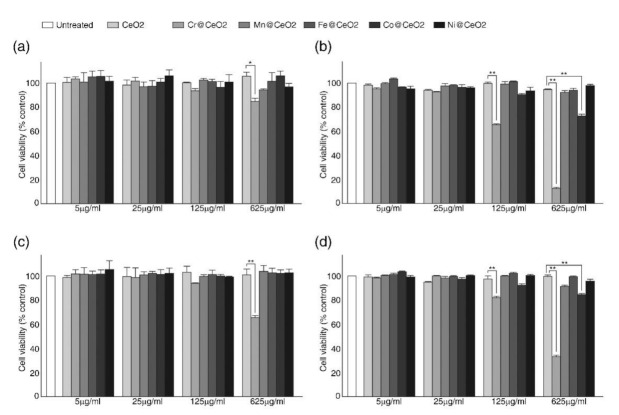
Transition metal doping increased the toxicity of cerium oxide nanoparticles (CeO_2_ NPs) in a dopant-dependent manner. HaCaT cells were incubated with undoped CeO_2_ or indicated TM@CeO_2_ NPs (5 to 625 μg/mL) for 24 (**a**,**c**) and 72 h (**b**,**d**). Cell viabilities were assessed and quantified using (**a**,**b**) MTT (3-(4,5-dimethylthiazol-2-yl)-2,5-diphenyltetrazolium bromide) and (**c**,**d**) NRU (neutral red uptake) assays, as described in Materials and Methods. Shown are representative data of at least three independent experiments. Mean ± SD. * *p* < 0.05, ** *p* < 0.01.

We next conducted MTT (3-(4,5-dimethylthiazol-2-yl)-2,5-diphenyltetrazolium bromide) and NRU (neutral red uptake) assays to assess the effects of transition metal doping on cell viabilities using three different cell lines, that is, HaCaT human keratinocytes, HEK293T cells (a human embryonic kidney cell line), and C3H10T1/2 mouse mesenchymal stem cells, respectively (Figure 2 and Figure S1). Consistent with previous studies, which showed CeO_2_ NPs were relatively non-toxic [51,52], the viability of HaCaT cells fed with undoped CeO_2_ NPs was comparable with that of untreated control cells even at a concentration of 625 μg/mL for up to 72 h (Figure 2). Furthermore, no significant decrease in viability was observed in cells treated with Mn-, Fe-, or Ni-doped CeO_2_ NPs for 24 and 72 h (Figure 2). In contrast, Co@CeO_2_ and Cr@CeO_2_ NPs exhibited significant toxicities (Figure 2). While exposure to Co@CeO_2_ NPs for 24 h had no significant effect on cell viabilities even at the highest concentration used (625 μg/mL) (Figure 2a,c), exposure to Co@CeO_2_ NPs at 625 μg/mL for 72 h reduced cell viability by ~30% (Figure 2b,d). Notably, exposure to Cr@CeO_2_ NPs caused a dose- and time-dependent decrease in viability (Figure 2). In HaCaT cells, exposure for 24 h resulted in modest but meaningful reductions (~7% at 125 μg/mL and ~15% at 625 μg/mL) and exposure for 72 h caused a further decreased the viability of HaCaT cells (~30% at 125 μg/mL and by >80% at 625 μg/mL) (Figure 2b,d). The viabilities of HEK293T cells were not significantly affected by exposure to relatively non-toxic NPs, but similar reductions were observed after exposure to Cr- or Co-doped NPs (Figure S1a–d). Interestingly, Co@CeO_2_ NPs, which showed modest but significant toxicity in both HaCaT and HEK293T cells, had no significant effect on the viability of C3H10T1/2 mouse mesenchymal stem cells, and only cells exposed to 625 μg/mL of Cr@CeO_2_ NPs for 72 h showed a reduction in viability of ~ 20%. These results indicated responsiveness to TM@CeO_2_ NPs is cell-type dependent (Figure S1e–h).

We next investigated whether differences in intracellular localization and cellular uptake efficiency predominantly determined dopant-dependent toxicity (Figure S2). Both relatively non-toxic Fe@CeO_2_ and toxic Cr@CeO_2_ NPs were readily internalized and localized in the perinuclear region of HaCaT cells (Figure S2a). Moreover, fluorescence-based cellular uptake assays revealed that uptake efficiencies of toxic Cr@CeO_2_ NPs were no higher than those of Fe@CeO_2_ NPs at 5~625 μg/mL after exposure up to 24 h (Figure S2b). Taken together, these data suggest that transition metal doping can affect the intrinsic toxicity of CeO_2_ NPs, and that doping with Cr or Co, dose- and time-dependently increases CeO_2_ nanoparticle toxicity.

### 2.2. Dopant-Dependent Toxicities of TM@CeO_2_ NPs Were Associated with Apoptotic Cell Death in HaCaT Cells

**Figure 3:**
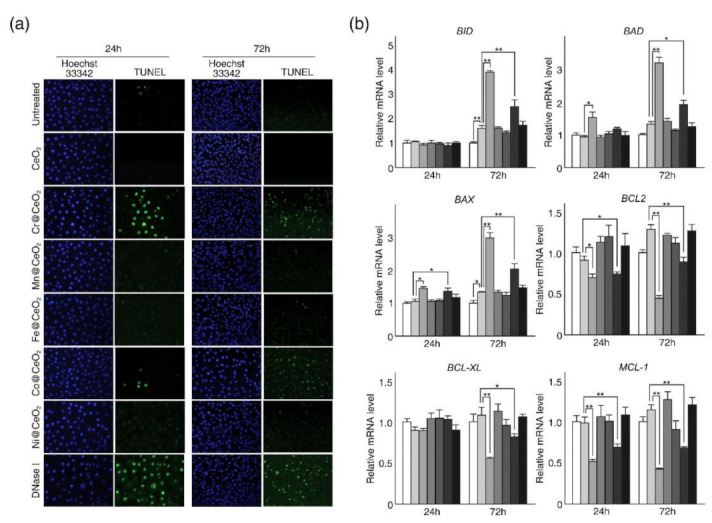
Exposure to toxic TM@CeO_2_ NPs led to apoptotic cell death in HaCaT cells. (**a**) Cells were exposed to 125 μg/mL of NPs (undoped CeO_2_ and TM@CeO_2_) for the indicated times (24 and 72 h). Shown are representative photomicrographic images of Terminal deoxynucleotidyl transferase dUTP nick end labeling (TUNEL) (green) and Hoechst 33342 (blue) double-stained cells (20×). Cells treated with DNase I were used as positive controls. (**b**) RT-qPCR analysis of genes involved in apoptotic cell death in untreated control and NPs treated cells. The total RNAs were isolated from untreated control, CeO_2_ NPs treated, and indicated TM@CeO_2_ NPs treated cells at the indicated times, and the relative mRNA levels of pro-apoptotic *BID* (BH3 interacting domain death agonist), *BAD* (BCL2 associated agonist of cell death), and *BAX* (BCL2 associated X) and anti-apoptotic *BCL2* (B-cell CLL/lymphoma 2), *BCL-XL* (BCL2 like 1), and *MCL-1* (Myeloid cell leukemia sequence 1) were measured by RT-qPCR. The mRNA levels of indicated genes were first normalized to the mRNA level of *GAPDH* (Glyceraldehyde-3-phosphate dehydrogenase), and data are presented as ratios of mRNA levels in NPs treated cells to mRNA levels in untreated cells at each time point (24 and 72 h). The qPCR data shown are representative of at least three independent experiments and are presented as mean ± SD (n = 3~5). * *p* < 0.05, ** *p* < 0.01.

We next investigated whether the observed decreases in cell viability were associated with apoptotic cell death (Figure 3). HaCaT cells were used for the in vitro analysis because they are derived from normal adult skin cells, and skin is one of the primary tissues affected by NPs. In addition, we used NPs at 125 μg/mL as both toxic and non-toxic NPs resulted in comparable viabilities at this concentration after 24 h but differences in viability after incubation for 72 h (Figure 2 and Figure S1). Terminal deoxynucleotidyl transferase dUTP nick end labeling (TUNEL) assays revealed extensive and prolonged DNA fragmentation in cells treated with toxic Cr@CeO_2_ NPs but lesser effects in cells exposed to Co@CeO_2_ NPs, no significant fragmentation in cells fed with undoped CeO_2_ or relatively non-toxic TM@CeO_2_ NPs (TM = Mn, Fe, and Ni) (Figure 3a). RT-qPCR analysis confirmed increased expressions of pro-apoptotic sensor genes *BID* (BH3 interacting domain death agonist) and *BAD* (BCL2 associated agonist of cell death) and the pro-apoptotic effector gene *BAX* (BCL2 associated X) in cells exposed to 125 μg/mL of Cr@CeO_2_ or Co@CeO_2_ NPs after 72 h (Figure 3b). Conversely, mRNA levels of the anti-apoptotic genes *BCL-2* (B-cell CLL/lymphoma 2), *BCL-XL* (BCL2 like 1), and *MCL-1* (Myeloid cell leukemia sequence 1) were markedly decreased in cells treated with Cr- or Co-doped CeO_2_ NPs (Figure 3b). Notably, while no significant changes in pro- and anti-apoptotic gene expressions were observed in HaCaT cells exposed to undoped CeO_2_ or relatively non-toxic TM@CeO_2_ NPs after 24 h, prolonged exposure (72 h) resulted in modest but meaningful increases in pro-apoptotic gene expressions in cells (Figure 3b). These data indicate that decreases in cell viability by toxic TM@CeO_2_ NPs (TM = Cr or Co) are at least in part due to apoptotic cell death.

### 2.3. Effect of Transition Metal Doping on Intracellular ROS Generation

**Figure 4:**
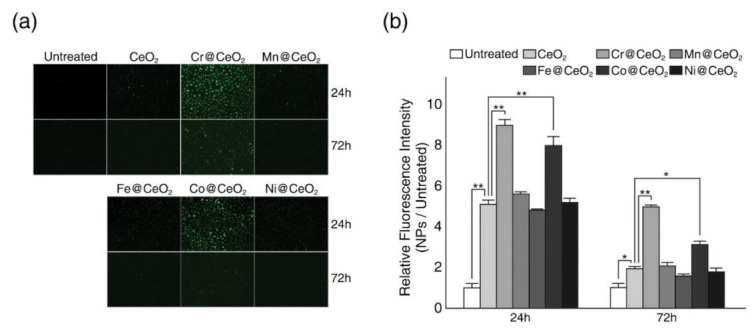
Effect of transition metal doping on intracellular reactive oxygen species (ROS) generation. Both undoped and TM@CeO_2_ NPs cause increased intracellular ROS generation in HaCaT cells, but exposure to toxic TM@CeO_2_ NPs (TM = Cr, Co) induced higher ROS levels. (**a**) Cells were treated with 125 μg/mL of NPs (undoped CeO_2_ and TM@CeO_2_) for the indicated times (24 and 72 h), and intracellular ROS levels were monitored using H2DCFDA, as described in Materials and Methods. Shown are representative photomicrographic images for intracellular ROS generation (20×). (**b**) ROS levels were quantified by measuring fluorescence intensity. Results are presented as the ratios of fluorescence intensities after NP treatment to fluorescence intensities of untreated cells. Shown are representative data of at least three independent experiments (n = 3~6). Mean ± SD. * *p* < 0.05, ** *p* < 0.01.

Since exposure to MONPs often causes oxidative stress, such as intracellular ROS generation, and these stresses are believed to be major factors of NP toxicity, we next examined the effect of transition metal doping on intracellular ROS generation (Figure 4). Surprisingly, we found that HaCaT cells exposed to NPs generated more ROS than untreated cells regardless of toxicity (Figure 4). However, levels of ROS measured in cells exposed to toxic TM@CeO_2_ NPs (TM = Cr, Co) were significantly greater than levels in undoped CeO_2_ NPs, whereas exposure to relative non-toxic TM@CeO_2_ NPs (TM = Fe, Mn, Ni) resulted in the ROS level similar to those observed in undoped NPs (Figure 4a, b). Levels of intracellular ROS appeared to decrease after 72 h, but HaCaT cells treated with toxic TM@CeO_2_ NPs (TM = Cr or Co) maintained higher ROS levels than those treated with relatively non-toxic NPs (Figure 4b). Considering that all tested NPs increased intracellular ROS generation but only Cr- and Cr-doped CeO_2_ NPs showed discernible cytotoxicity, these results suggest that either ROS level or the ability of cells to respond to ROS more critically determine NPs-mediated toxicity than oxidative stress itself.

### 2.4. Dopant-Dependent Toxicity Was Associated with a Failure of Cells to Activate NRF2 Expression

**Figure 5:**
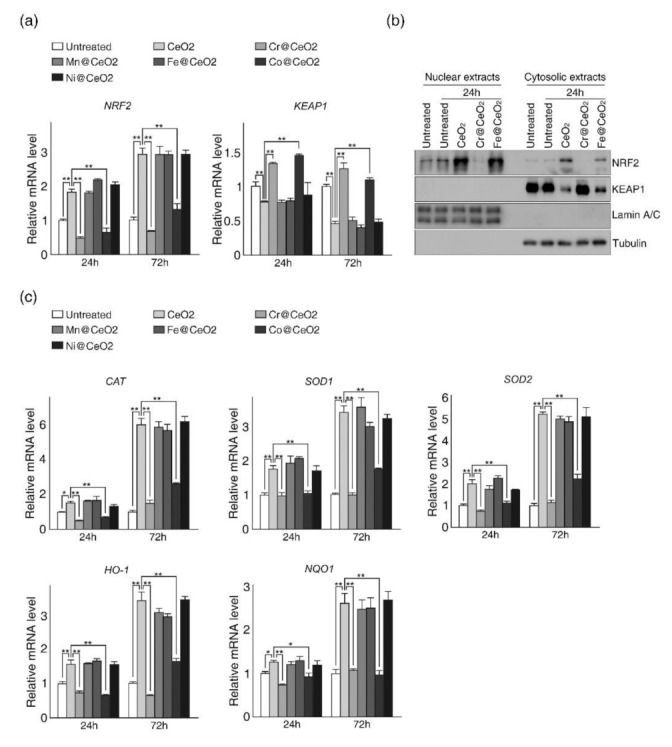
Toxic TM@CeO_2_ NPs (TM = Cr, Co) failed to activate the NRF2 dependent antioxidant defense system. (**a**,**b**) HaCaT cells treated with Cr@CeO_2_ or Co@CeO_2_ NPs failed to activate NRF2 expression. (**a**) RT-qPCR analysis of *NRF2(nuclear factor erythroid 2-related factor 2)* and *KEAP1* (kelch like ECH associated protein 1) genes in non-treated controls and NPs-treated cells. The total RNAs were isolated from untreated control, CeO_2_ NPs-treated, and TM@CeO_2_ NPs-treated cells after the indicated treatment time and relative mRNA levels were measured by RT-qPCR. (**b**) Immunoblot analysis of NRF2 and KEAP1 before and after NPs exposure. Nuclear and cytosolic extracts were prepared from cells treated or not with NPs for 24 h and subjected to immunoblot analysis to detect NRF2, KEAP1, Lamin A/C, and Tubulin. Lamin A/C and Tubulin were used as controls for nuclear and cytosolic fractions, respectively. (**c**) RT-qPCR analysis of target genes of NRF2 in control and NPs-treated cells. Relative mRNA levels of *CAT* (catalase), *SOD1* (superoxide dismutase 1, cytosol), *SOD2* (superoxide dismutase 2, mitochondria), *HO-1* (heme oxygenase 1), and *NQO1* (NAD(P)H quinone dehydrogenase 1) were measured using cDNA prepared from the same cells used in (**a**). The mRNA levels of indicated genes (**a**,**c**) were normalized to mRNA level of *GAPDH*, and data are presented as ratios of mRNA levels in NP-treated cells to those in untreated cells at each time point (24 and 72 h). The qPCR results are representative of at least three independent experiments and presented as mean ± SD (n = 3~5). * *p* < 0.05, ** *p* < 0.01.

Because oxidative stresses induced by reactive oxidants are mainly countered by the NRF2-KEAP1 signaling pathway (a major antioxidant defense system), we investigated whether ROS increases by CeO_2_ or TM@CeO_2_NPs led to the activation of this pathway (Figure 5). RT-qPCR analysis revealed increased expression of *NRF2* and decreased expression of *KEAP1* (a negative regulator of NRF2) in HaCaT cells treated with undoped CeO_2_ NPs and similar results in cells treated with relatively non-toxic TM@CeO_2_ NPs (TM = Mn, Fe, or Ni) (Figure 5a). Surprisingly, no significant increase in *NRF2* mRNA level and decrease in *KEAP1* mRNA level was observed in cells exposed to toxic TM@CeO_2_ NPs (TM = Cr or Co) despite elevated intracellular ROS levels (Figure 4 and Figure 5a). Immunoblot analysis confirmed increased NRF2 levels in nuclear and cytosolic fractions and decreased KEAP1 levels after exposing cells to relatively non-toxic CeO_2_ or Fe@CeO_2_ NPs, but not in cells exposed to toxic Cr@CeO_2_ NPs (Figure 5b and Figure S3). Next, we examined the expression of downstream target genes of NRF2, which include *CAT* (catalase), *SOD1* (superoxide dismutase 1, cytosol), *SOD2* (superoxide dismutase 2, mitochondria), *HO-1* (heme oxygenase 1), and *NQO1* (NAD(P)H quinone dehydrogenase 1) (Figure 5c). As was expected, the expression of NRF2 target genes was markedly increased in HaCaT cells exposed to relatively non-toxic NPs but not in cells exposed to toxic TM@CeO_2_ NPs (Figure 5c). These observations suggest that intracellular ROS increases induced by relatively non-toxic NPs can be countered in cells, at least in part, by activation of the antioxidant defense system mediated by NRF2, and that the failure of cells to cope with elevated ROS levels underlies the dopant-dependent toxicity of CeO_2_ NPs.

### 2.5. Dopant Dependent Toxicity Was Associated with H3K4me3 and H3K27me3 Modification at NRF2 Promoter

**Figure 6:**
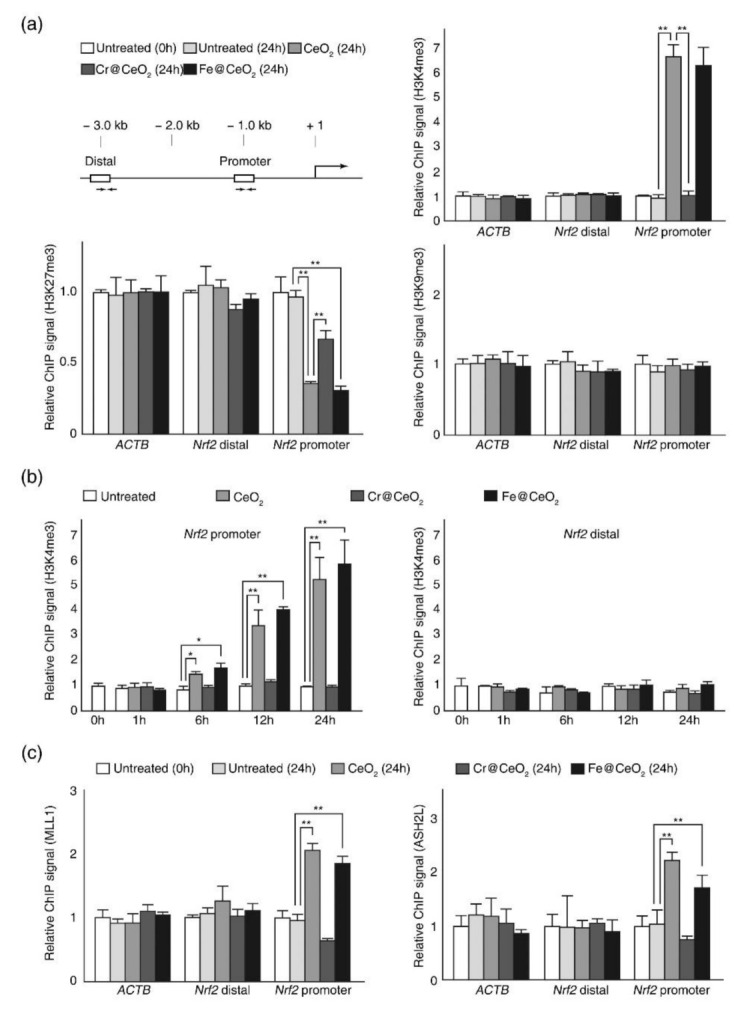
Dopant-dependent TM@CeO_2_ toxicity was associated with dynamic changes in histone lysine modifications. (**a**) CeO_2_ and Fe@CeO_2_ NPs, but not toxic Cr@CeO_2_ NPs, increased H3K4me3 but decreased H3K27me3 level at the promoter of *NRF2* gene. (upper left) Schematic representation of the *NRF2* gene with amplicons (promoter and distal regions) analyzed by chromatin immunoprecipitation (ChIP)-qPCR. Chromatins prepared from the cells before (0 h) and after (24 h) NPs exposure were precipitated with α-H3K4me3, α-H3K9me3, or α-H3K27me3 antibodies. qPCR analysis was performed to assess the enrichment of modified histones at the promoter and distal regions of the *NRF2* gene. (**b**) Time course ChIP analysis for H3K4 trimethylation induced by NPs. Chromatins were prepared from HaCaT cells exposed to NPs for the indicated times and precipitated with α-H3K4me3 antibodies. (**c**) Binding of MLL (mixed-lineage leukemia) complex at *NRF2* promoter increased after non-toxic NPs treatment but not after treatment with toxic Cr@CeO_2_ NPs. Chromatins were prepared as described in (**a**) and precipitated with α-MLL1 (left) or α-ASH2L (right) antibodies. For each chromatin, ChIP using IgG was performed to check chromatin quality. qPCR analyses shown in (**b**,**c**) were performed as in (**a**). For the relative ChIP signal, the % input (indicated antibody) was calculated for all samples, and data are presented as ratios of % input (indicated antibody) in NP-treated cells to those in untreated control cells. qPCR data are representative of at least three independent experiments and are presented as mean ± SD (n = 3~5). * *p* < 0.05, ** *p* < 0.01.

Since lysine methylation of core histones is known to be involved in both activation and repression of genes depending on the site and status of modification [53], we next investigated whether the failure of NRF2 expression following exposure to toxic TM@CeO_2_ NPs was associated with changes in histone lysine methylation (Figure 6a, b). Chromatin immunoprecipitation (ChIP) assays revealed that the exposure of HaCaT cells to undoped CeO_2_ or Fe@CeO_2_ NPs resulted in significant increases in the trimethylation of histone H3 lysine 4 (H3K4me3) and a discernible decrease in the trimethylation of histone H3 lysine 27 (H3K27me3) at *NRF2* promoter (Figure 6a, upper right and lower left panel). However, exposure to toxic Cr@CeO_2_ NPs had little effect on H3K4me3 or H3K27me3 at the promoter (Figure 6a). Interestingly, trimethylation of histone H3 lysine9, which also marks repressed gene expression, was not affected by NPs exposure (Figure 6a, lower right panel). Because levels of histone methylation are determined by methylation and demethylation, we conducted a time course chromatin immunoprecipitation analysis to confirm that exposure to Cr@CeO_2_ NPs did not promote H3K4me3 demethylation. As shown in Figure 6b, H3K4me3 level at the promoter of *NRF2* gradually increased after exposure to undoped CeO_2_ or Fe@CeO_2_ NPs for up to 24 h, but no discernible change in H3K4me3 level was detected after treatment with Cr@CeO_2_ NPs for the same time. Finally, we examined the binding of the MLL1 (mixed-lineage leukemia 1) complex (a major histone lysine methylase for H3K4 trimethylation) at *NRF2* gene. As was expected, exposure to relatively non-toxic NPs but not to toxic Cr@CeO_2_ NPs increased bindings of MLL1 and ASH2L (a key component of MLL1 complex) at *NRF2* promoter (Figure 6c). Taken together, these data strongly suggest that oxidative stresses induced by CeO_2_ and relatively non-toxic TM@CeO_2_ NPs can be countered by transcriptional activation of NRF2 via dynamic changes in H3K4me3and H3K27me3, and that failure of NRF2 activation is an underlying cause of the dopant-dependent toxicity of TM@CeO_2_ NPs.

In conclusion, our current study shows that TM@CeO_2_ NPs could exhibit dopant-dependent toxicity. Cr was the most toxic dopant among the transition metal tested, and Fe, Mn, or Ni appeared to have no significant effect on the intrinsic toxicity of CeO_2_ NPs. In particular, our data support the idea that activation of NRF2 signaling pathway rather than oxidative stress per se critically determines NPs-mediated toxicity, as all tested CeO_2_ NPs elevated intracellular ROS levels but only the relatively non-toxic NPs induced intracellular antioxidant defense mechanism at least in part by activating NRF2 expression. In addition, our observations of dynamic changes in H3K4me3 and H3K27me3 histone modifications and increased binding of MLL1 complex at the *NRF2* promoter following NPs exposure suggest MLL1 complex participates in the regulation of NRF2 expression, which we hope provides new insights into the molecular mechanism responsible for activating NRF2 dependent antioxidant defense system. Lastly, it should be noted that despite the observed relatively non-toxic natures of undoped CeO_2_ and Fe-, Mn-, and Ni-doped CeO_2_ NPs, the safety of these NPs with respect to long-term exposure remains undetermined, and thus, the study emphasizes the importance of carefully evaluating engineered NPs for biological safety before they are adopted for industrial and biomedical purposes.

## 3. Materials and Methods

### 3.1. Preparation of Transition Metal-Doped CeO_2_ NPs

CeO_2_ NPs were synthesized using a modified thermal method [25,26]. For transition metal doping, precursor solutions were prepared by one-pot synthesis. The desired amount (1 mol%) of each TM dopant (Cr, Mn, Fe, Co, and Ni) in the form of TM(NO_3_)_3_∙9H_2_O (99% purity) was added to each synthetic gel solution with stirring until the solution became homogeneous and transparent. The solution was then transferred to a Teflon-lined autoclave and heated at 220 °C for 10 h in a convection oven. The resulting CeO_2_ and TM@CeO_2_ NPs were filtered and washed with deuterium-depleted water (DDW) to remove residues. All substances used for doping were purchased from Sigma–Aldrich (Sigma, St Louis, MO, USA).

### 3.2. Characterization of TM@CeO_2_ NPs

The structures of fabricated CeO_2_ NPs and the five TM@CeO_2_ NPs were analyzed by using a JEM-3010 high-resolution transmission electron microscopy (HR-TEM, JEOL, Tokyo, Japan) at 300 kV and X-ray diffraction (XRD) patterns were obtained using Ni-filtered Cu–Kα radiation from a D8 Advance diffractometer (Bruker, Karlsruhe, Germany).

### 3.3. Cell Culture and NPs Exposure

HaCaT human keratinocytes were kindly provided by Dr. S. Kwon (Inha University, Korea). Cells were maintained in Dulbecco’s modified Eagle’s medium (DMEM, WelGENE, Gyeongsan, Korea) supplemented with 10% fetal bovine serum (FBS, WelGENE, Gyeongsan, Korea) and 1% penicillin–streptomycin (GE Healthcare, Madison, WI, USA) in a humidified atmosphere with 5% CO_2_ at 37 °C. For NPs exposure, 10 mg/mL of TM@CeO_2_ NPs in DMEM supplemented with 10% FBS were prepared using a vortex mixer to prevent aggregation and then added to culture plates at the final concentrations of 5, 25, 125, or 625 μg/mL.

### 3.4. Cell Viability Assays

The effects of TM@CeO_2_ NPs on cell viability were assessed by MTT and NRU assays, as previously described [54,55]. Briefly, HaCaT, HEK293T, and C3H10T1/2 cells were seeded at 2 × 10^4^ cells per well in 96-well cell culture plates and cultured for 24 h. Cells were then exposed to undoped CeO_2_ or TM@CeO_2_ NPs for 24 or 72 h. For MTT assays, cells were washed twice with phosphate-buffered saline (PBS, GIBCO, Grand Island, NY, USA), and then MTT solution (Sigma, St Louis, MO, USA) was added to each well to a final concentration of 0.5 mg/mL. One hour later, formazan crystals that formed were dissolved in 50% dimethyl sulfoxide (DMSO, Sigma, St Louis, MO, USA)/50% methanol (Merck, Darmstadt, Germany). For NRU assays, cells were washed twice with PBS following exposure to NPs for 24 or 72 h and then incubated for 4 h in OPTI-MEMI (GIBCO, Grand Island, NY, USA) containing 40 ng/mL of neutral red reagent (Sigma, St Louis, MO, USA). After incubation, wells were eluted with 50% ethanol (Merck, Darmstadt, Germany)/1% glacial acetic acid (Merck, Darmstadt, Germany). Absorbances were measured using an XFluor4 microplate reader (Tecan, Männedorf, Switzerland) at 595 nm for MTT assays and 540 nm for NRU assays. Cell viabilities were expressed as percentages of control cells using {(O.D. sample − O.D. blank)/(O.D. control − O.D. blank) × 100}.

### 3.5. TUNEL Assay

Terminal deoxynucleotidyl transferase dUTP nick end labeling (TUNEL) assays were performed using the In Situ Cell Death Detection Kit, Fluorescein (Roche, Mannheim, Germany). HaCaT cells were seeded at 2 × 10^4^ cells per well in a 96-well cell culture plate and 24 h later, exposed to 125 μg/mL of undoped CeO_2_ or TM@CeO_2_ NPs for 24 or 72 h. Following fixation with 4% paraformaldehyde (EMS, Hatfield, PA, USA) for 30 min and permeabilization with 0.1% Triton X-100 (Merck, Darmstadt, Germany) in PBS for 10 min, cells were incubated with TUNEL reaction solution for 1 h at 37 °C in the dark and stained with Hoechst 33342 (Invitrogen, Carlsbad, CA, USA) for 5 min. Images were taken at 20× magnification using an Olympus IX71 inverted microscope equipped with a U-RFL-T mercury lamp (Olympus, Tokyo, Japan). Cells treated with 1,000 units/mL of DNase I (Promega, Madison, WI, USA) for 10 min were used as positive controls.

### 3.6. Measurement of Intracellular Reactive Oxygen Species (ROS) Levels

Intracellular ROS levels were measured using dichlorofluorescein diacetate oxidation, as previously described [56]. Cells were seeded at 2 × 10^5^ cells per well in 6-well plates, incubated for 24 h, and exposed to 125 μg/mL of undoped CeO_2_ or TM@CeO_2_ NPs for 24 or 72 h. Following exposure, cells were washed with PBS and incubated with 50 μM of 2′,7′-dichlorofluorescin diacetate (DCFDA, Invitrogen, Carlsbad, CA, USA) for 30 min. Images were taken using an Olympus IX71 inverted microscope equipped with a U-RFL-T mercury lamp at excitation wavelengths of 488 nm and processed using Adobe Photoshop CC2018 software (Adobe Systems, San Jose, CA, USA). To quantify ROS levels, fluorescence intensities were measured using a Synergy HTX multi-mode microplate reader (Bio-Tek, Winooski, VT, USA) and Gen5 software (Bio-Tek, Winooski, VT, USA) at excitation and emission wavelengths of 485 nm and 528 nm, respectively. Relative fluorescence intensity was presented as a ratio of (O.D. sample − O.D. blank) to (O.D. untreated − O.D. blank).

### 3.7. Cell Fractionation and Immunoblot Analysis

HaCaT cells were washed with PBS and lysed with hypotonic buffer (10 mM HEPES pH 7.9, 10 mM KCl, 0.1 mM ethylenediaminetetraacetic acid (EDTA) pH 8.0, and 0.3% NP-40) in the presence of a protease inhibitor cocktail (Roche, Mannheim, Germany). Lysates obtained were passed through a 26G1/2 needle 10 times, incubated on ice for 10 min, and then centrifuged at 5,000× *g* for 10 min. Supernatants were used as a cytosolic fraction, and nuclear fractions were prepared by suspending pellets in radioimmunoprecipitation assay (RIPA) buffer (10 mM Tris-HCl, pH 7.5, 1 mM EDTA, pH 8.0, 150 mM NaCl, 1% Triton X-100, 1% sodium deoxycholate, and 0.1% sodium dodecyl sulfate (SDS)), sonicating at 40% amplitude for 5 × 30 s using a VCX130 sonicator (Sonics, Newtown, CT, USA), and then centrifugation at 13,000× *g* for 20 min. Immunoblot analysis was performed using a standard protocol. Detailed information regarding antibodies and working concentrations is provided in Supplementary Material (Table S1).

### 3.8. Quantitative RT-PCR Analysis (RT-qPCR)

For RT-qPCR analysis, total RNA was isolated using an RNeasy plus mini kit (QIAGEN, Hilden, Germany), and cDNA was synthesized using a GoScript reverse transcription system (Promega, Madison, WI, USA), according to the manufacturer’s instructions. Quantitative PCR was conducted using a QuantStudio 1 Real-Time PCR system (ThermoFisher Scientific, Waltham, MA, USA) using SYBR Green I (Invitrogen, Carlsbad, CA, USA) and i-StarTaq DNA polymerase (Intron, Sungnam, Korea). mRNA levels were normalized to *GAPDH* mRNA, and data are presented as indicated in Figure 3 and Figure 5. Primer sets used are detailed in Supplementary Materials (Table S2).

### 3.9. Chromatin Immunoprecipitation (ChIP)–qPCR Analysis

ChIP assays were performed as previously described [57]. Briefly, 100~300 µg of sonicated chromatins were precleared for 2 h using protein A/G sepharose 4 Fast Flow (GE Healthcare, Madison, WI, USA) in the presence of 4 mg/mL salmon sperm DNA (Invitrogen, Carlsbad, CA, USA) and 0.5 mg/mL bovine serum albumin (Sigma, St Louis, MO, USA) and then subjected to immunoprecipitation using appropriate antibodies. Purified DNA obtained was analyzed by quantitative PCR (qPCR) using a QuantStudio 1 Real-Time PCR system. For quantification, the % input value per sample was calculated, and the data are presented as relative ChIP signals as indicated in Figure 6. The antibodies and primers used for ChIP-qPCR analysis are listed in Tables S1 and S3.

### 3.10. Statistical Analysis

Results of cell viability assays and all qPCR-based experiments are representative of at least three independent experiments (as indicated in the figure legends) and are presented as the means ± SDs. Statistical significance and *p*-values were determined by two-tailed *t*-tests of the indicated paired groups using Microsoft Excel (version 2102, Microsoft, Redmond, WA, USA). Differences were considered significant when *p*-values were < 0.05.

## Data Availability

The data presented in this study are available on request from the corresponding author.

## Supplementary Materials

The following are available online at https://www.mdpi.com/1422-0067/22/6/3087/s1, Figure S1: Effect of transition metal doping on the viabilities of HEK293T and C3H10T1/2 cells. Figure S2: Intracellular localization and cellular uptake efficiency of toxic Cr@CeO_2_ NPs and relatively non-toxic Fe@CeO_2_ NPs. Figure S3: Exposure to relatively non-toxic undoped or Fe-doped CeO_2_ NPs led to increased NRF2 and decreased KEAP1 in HaCaT cells. Table S1: Information on the antibodies used in this study, Table S2: Information on the primers used for RT-qPCR, Table S3: Information on the primers used for ChIP-qPCR, References.
