# Supplementary material for: Dopant-Dependent Toxicity of CeO2 Nanoparticles Is Associated with Dynamic Changes in H3K4me3 and H3K27me3 and Transcriptional Activation of NRF2 Gene in HaCaT Human Keratinocytes
Source: Int J Mol Sci. 2021 Mar 17;22(6):3087. doi: 10.3390/ijms22063087 (PMC8002609; doi:10.3390/ijms22063087)
Supplement: Supplementary file 1 [file ijms-22-03087-s001.zip › Choi.Supplementary information.docx]

**Supplementary Information**


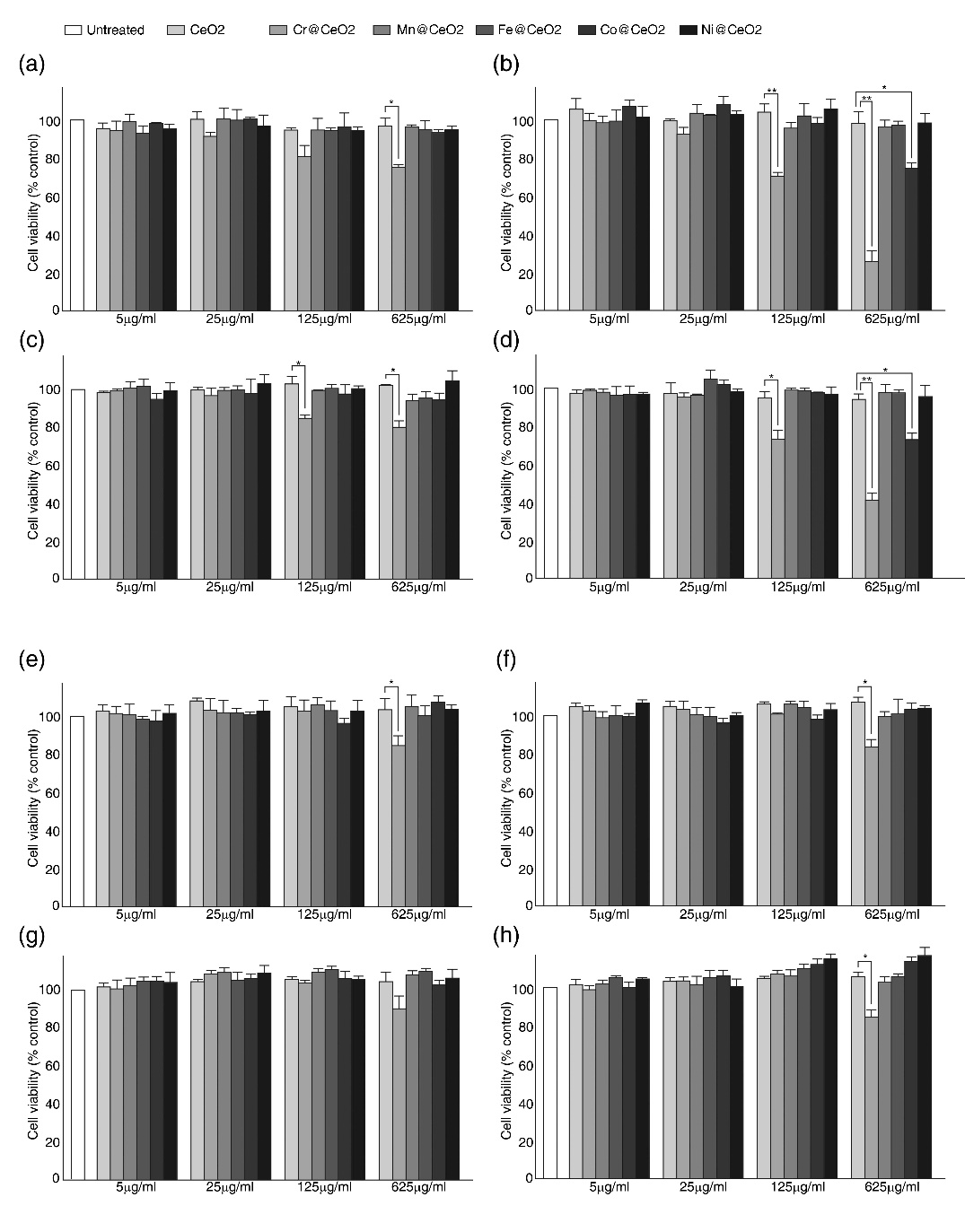


**Figure S1. Effect of transition metal doping on the viabilities of HEK293T and C3H10T1/2 cells.** Cells were incubated with undoped CeO_2_ or indicated TM@CeO_2_ NPs (5 to 625 μg/ml) for 24 (a, c, e, g) and 72 hours (b, d, f, h). Cell viabilities were assessed and quantified using MTT assay (a, b, e, f) and NRU assay (c, d, g, h) as described in materials and methods. Shown are representative data of at least three independent experiments. Mean ± SD. *p < 0.05, **p < 0.01.


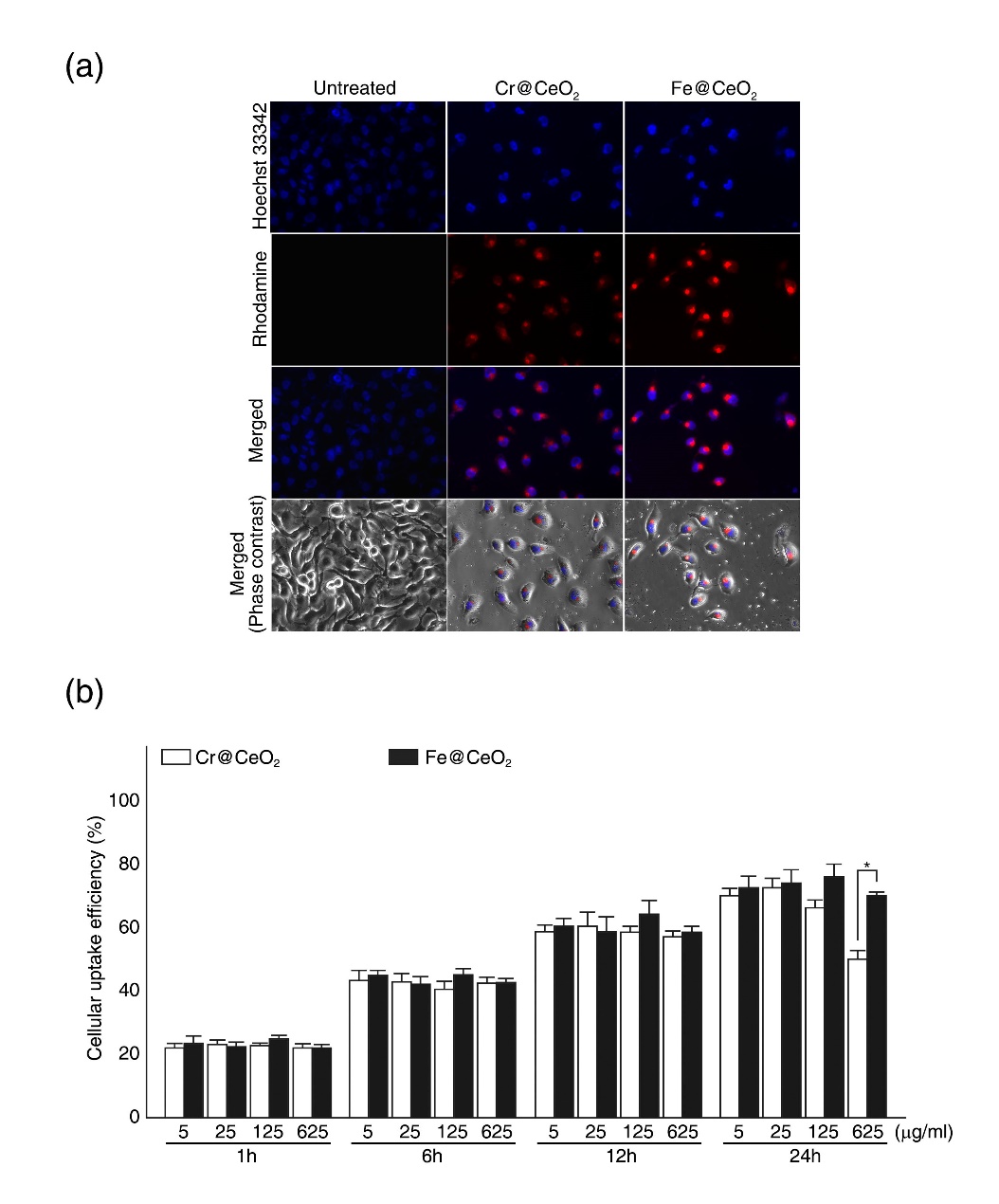


**Figure S2. Intracellular localization and cellular uptake efficiency of toxic Cr@CeO_2_ NPs and relatively non-toxic Fe@CeO_2_ NPs.** (a) Both toxic and relatively non-toxic TM@CeO_2_ NPs are internalized and localized at perinuclear region. To determine the intracellular localization of NPs, HaCaT cells were grown to 80 % confluence and then exposed to rhodamine-labeled NPs for a final concentration of 25 μg/ml for 24 hours. Cells were fixed with 4 % paraformaldehyde for 10 minutes at RT, stained with 5 μg/ml of Hoechst 33342 (Thermo) for 5 minutes, and intracellular NP localizations were observed using an Olympus IX71 inverted microscope equipped with a U-RFL-T (Olympus) mercury lamp and a TH4-200 (Olympus) photosystem (32🞨). Images were processed using Adobe Photoshop CC2018 software. (b) Cellular uptake assays for toxic and relatively non-toxic NPs in HaCaT cell. Quantification of cellular uptake efficiency was performed as previously described with modifications [1]. Cells were seeded at 1 x 10^4^ cells per well into 96-well plates and cultured overnight. The cells were treated with rhodamine-labeled NPs at a concentration of 5 to 625 μg/ml for indicated time periods. After incubation, the cells were washed with cold PBS three times and lysed by 0.5 % Triton X-100 in 0.2 N NaOH. the fluorescence intensity of each sample was measured by a Synergy HTX multi-mode microplate reader using Gen5 software at an excitation and emission wavelengths of 530 nm and 590 nm, respectively. Cellular uptake efficiency was presented as a ratio (O.D. sample) to (O.D. total). The total amount was obtained by measuring the fluorescence intensity of rhodamine present in the feed nanoparticle suspension immediately upon NPs exposure.


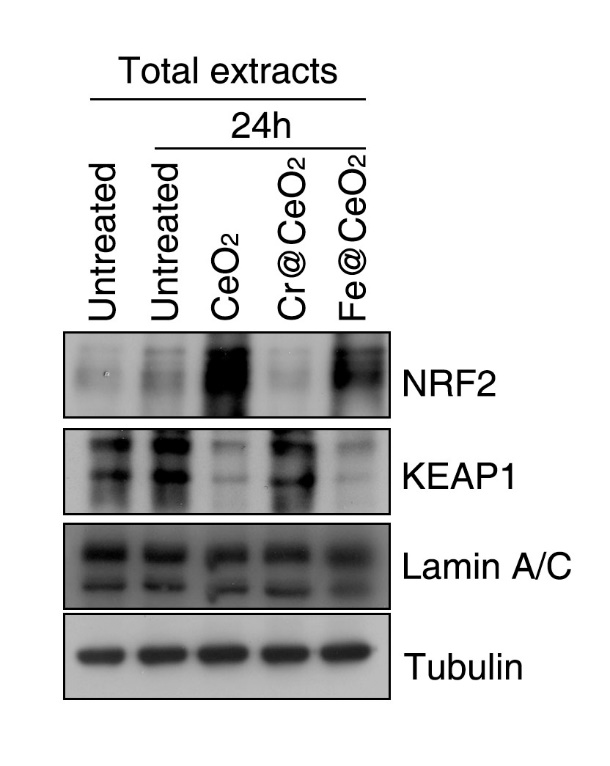


**Figure S3. Exposure to relatively non-toxic undoped or Fe-doped CeO2 NPs led to increased NRF2 and decreased KEAP1 in HaCaT cells.** Total extracts were prepared from cells treated or not with NPs for 24 hours and subjected to immunoblot analysis to detect NRF2, KEAP1, Lamin A/C, and Tubulin. Lamin A/C and Tubulin were used as loading controls.

**Table S1. Information on the antibodies used in this study**

| Antibody | Source | Identifier | Application |
| --- | --- | --- | --- |
| NRF2 | Proteintech | 16396-1-AP;  RRID: AB_2782956 | WB (1/2,000) |
| KEAP1 | Cell signaling | 8047  RRID: AB 10860776 | WB (1/2000) |
| MLL1 | Bethyl | A300-086A;  RRID: AB_242510 | ChIP (4ug) |
| Ash2l | Bethyl | A300-489A;  RRID: AB_451024 | ChIP (4ug) |
| Lamin A/C (N-18) | SantaCruz | sc-6215;  RRID: AB_648152 | WB (1/10,000) |
| Tubulin (1A9) | SantaCruz | sc-134230;  RRID: AB_2272485 | WB (1/10,000) |
| H3K4me3 | Abcam | ab8580;  RRID: AB_306649 | ChIP (4ug) |
| H3K9me3 | Abcam | ab8898;  RRID: AB_306848 | ChIP (4ug) |
| H3K27me3 | Abcam | ab6002;  RRID: AB_305237 | ChIP (4ug) |
| Rabbit IgG | Vectashield | I-1000;  RRID: AB_2336355 | ChIP (4ug) |
| Mouse IgG-HRP | GE | NA-931V;  RRID: AB_772210 | WB (1/10,000) |
| Rabbit IgG-HRP | Jackson ImmunoResearch | 711-035-152;  RRID: AB_10015282 | WB (1/10,000) |
| Goat IgG-HRP | Jackson ImmunoResearch | 705-035-147  RRID: AB 2313587 | WB (1/10,000) |

**Table S2. Information on the primers used for RT-qPCR**

| **Gene** | **Forward primer** | **Reverse primer** | **Source** |
| --- | --- | --- | --- |
| *NRF2* | ATAGCTGAGCCCAGTATC | CATGCACGTGAGTGCTCT | [2] |
| *KEAP1* | CCTTCAGCTACACCCTGGAG | AACATGGCCTTGAAGACAGG | [3] |
| *SOD1* | AGGGCATCATCAATTTCGAG | ACATTGCCCAAGTCTCCAAC | [4] |
| *SOD2* | TTGGCCAAGGGAGATGTTAC | AGTCACGTTTGATGGCTTCC | [5] |
| *CAT* | GCCTGGGACCCAATTATCTT | GAATCTCCGCACTTCTCCAG | [6] |
| *HO-1* | CTGAGTTCATGAGGAACTTTCAGAAG | TGGTACAGGGAGGCCATCAC | [7] |
| *NQO1* | GGGCAAGTCCATCCCAACTG | GCAAGTCAGGGAAGCCTGGA | [8] |
| *BCL2* | GATAACGGAGGCTGGGATGC | TCACTTGTGGCCCAGATAGG | [9] |
| *BCL-XL* | CCTGCCTGCCTTTGCCTAA | TGGGCTCAACCAGTCCATTG | [9] |
| *MCL-1* | AAGAGGCTGGGATGGGTTTG | CAGCAGCACATTCCTGATGC | [9] |
| *BAD* | CCTCAGGCCTATGCAAAA | AAACCCAAAACTTCCGATGG | [10] |
| *BID* | AGCACAGTGCGGATTCTGTC | ACCGTTGTTGACCTCACAGT | [9] |
| *BAX* | ACTCCCCCCGAGAGGTCTT | GCAAAGTAGAAAAGGGCGACAA | [11] |
| *GAPDH* | GAAGGTGAAGGTCGGAGT | GAAGATGGTGATGGGATTTC |  |

**Table S3. Information on the primers used for ChIP-qPCR**

| **Gene** | **Forward primer** | **Reverse primer** | **Source** |
| --- | --- | --- | --- |
| *ACTB* | CACCCAGCACATTTAGCTAGCTGA | TTCAGAGCAACTGCCCTGAAAGCA | [12] |
| *NRF2*  promoter | AGAGAAAGTAAGCTCTGCAGC | CTGGCAGTGGTTTTGCTATTT | [13] |
| *NRF2*  distal | ACTTTTCCCATGGCCTCTCT | CTTGAAAAAGCCCTGACAGC | In this study |

**References**

[1] Zeng, X.; Tao, W.; Mei, L.; Huang, L.; Tan, C.; Feng, S. S. Cholic acid-functionalized nanoparticles of star-shaped PLGA-vitamin E TPGS copolymer for docetaxel delivery to cervical cancer. *Biomaterials*, **2013**,34(25), 6058–6067.

[2] Cho, J. M.; Manandhar, S.; Lee, H. R.; Park, H. M.; Kwak, M. K. Role of the Nrf2-antioxidant system in cytotoxicity mediated by anticancer cisplatin: implication to cancer cell resistance. *Cancer Lett.,* **2008**,*260*(1-2), 96–108.

[3] Tanigawa, S.; Fujii, M.; Hou, D. X. Action of Nrf2 and Keap1 in ARE-mediated NQO1 expression by quercetin. *Free Radic. Biol. Med.,* **2007,** 42(11):1690-1703.

[4] Radak, Z.; Bori, Z.; Koltai, E.; Fatouros, I. G.; Jamurtas, A. Z.; Douroudos, I. I.; Terzis, G.; Nikolaidis, M. G.; Chatzinikolaou, A.; Sovatzidis, A.; Kumagai, S.; Naito, H.; Boldogh, I. Age-dependent changes in 8-oxoguanine-DNA glycosylase activity are modulated by adaptive responses to physical exercise in human skeletal muscle. *Free Radic. Biol. Med.,* **2011**, 51(2), 417–423.

[5] Cho, J. H.; Kim, M. J.; Kim, K. J.; Kim, J. R. POZ/BTB and AT-hook-containing zinc finger protein 1 (PATZ1) inhibits endothelial cell senescence through a p53 dependent pathway. *Cell Death Differ.,* **2012**, 19(4), 703–712.

[6] Yoon, J. H.; Kang, Y. H.; Choi, Y. J.; Park, I. S.; Nam, S. W.; Lee, J. Y.; Lee, Y. S.; Park, W. S. Gastrokine 1 functions as a tumor suppressor by inhibition of epithelial-mesenchymal transition in gastric cancers. *J. Cancer Res. Clin. Oncol.* **2011**, 137(11), 1697–1704.

[7] Das, S.; Singh, S.; Dowding, J. M.; Oommen, S.; Kumar, A.; Sayle, T. X.; Saraf, S.; Patra, C. R.; Vlahakis, N. E.; Sayle, D. C.; Self, W. T.; Seal, S. The induction of angiogenesis by cerium oxide nanoparticles through the modulation of oxygen in intracellular environments. *Biomaterials,* **2012**, 33(31), 7746–7755.

[8] Lee, S.; Lim, M. J.; Kim, M. H.; Yu, C. H.; Yun, Y. S.; Ahn, J.; Song, J. Y. An effective strategy for increasing the radiosensitivity of Human lung Cancer cells by blocking Nrf2-dependent antioxidant responses. *Free Radic. Biol. Med.,* **2012**, 53(4), 807–816.

[9] Singh, S. K.; Banerjee, S.; Acosta, E. P.; Lillard, J. W.; Singh, R. Resveratrol induces cell cycle arrest and apoptosis with docetaxel in prostate cancer cells via a p53/ p21WAF1/CIP1 and p27KIP1 pathway. *Oncotarget,* **2017**, 8(10), 17216–17228.

[10] Lu, J.; Zhang, Q.; Tan, D.; Luo, W.; Zhao, H.; Ma, J.; Liang, H.; Tan, Y. GABA A receptor π subunit promotes apoptosis of HTR-8/SVneo trophoblastic cells: Implications in preeclampsia. *Int. J. Mol. Med.,* **2016,** 38(1), 105–112.

[11] Sun, X. X.; Wang, Y. G.; Xirodimas, D. P.; Dai, M. S. Perturbation of 60 S ribosomal biogenesis results in ribosomal protein L5- and L11-dependent p53 activation. *J. Biol. Chem.,* **2010**, 285(33), 25812–25821.

[12] Pajares, M.; Jiménez-Moreno, N.; García-Yagüe, Á. J.; Escoll, M.; de Ceballos, M. L.; Van Leuven, F.; Rábano, A.; Yamamoto, M.; Rojo, A. I.; Cuadrado, A. Transcription factor NFE2L2/NRF2 is a regulator of macroautophagy genes. *Autophagy,* **2016**, 12(10), 1902–1916.

[13] Kang, K. A.; Piao, M. J.; Ryu, Y. S.; Kang, H. K.; Chang, W. Y.; Keum, Y. S.; Hyun, J. W. Interaction of DNA demethylase and histone methyltransferase upregulates Nrf2 in 5-fluorouracil-resistant colon cancer cells. *Oncotarget,* **2016**, 7(26), 40594–40620.
