# Supplementary figures and images for: Dopant-Dependent Toxicity of CeO2 Nanoparticles Is Associated with Dynamic Changes in H3K4me3 and H3K27me3 and Transcriptional Activation of NRF2 Gene in HaCaT Human Keratinocytes
Source: Int J Mol Sci. 2021 Mar 17;22(6):3087. doi: 10.3390/ijms22063087 (PMC8002609; doi:10.3390/ijms22063087)

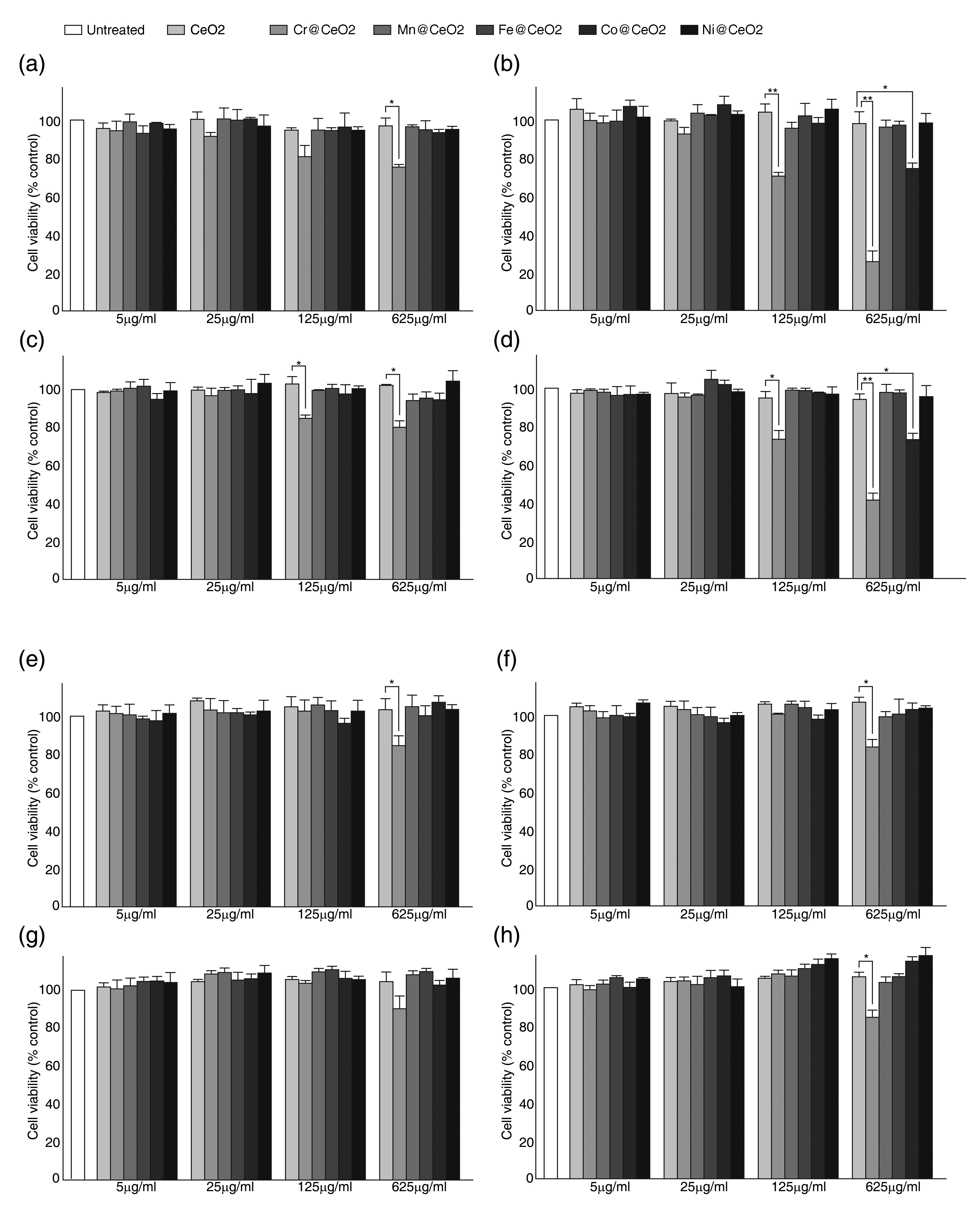

Supplement: Supplementary file 1 [file ijms-22-03087-s001.zip › Figure S1 (composite).jpg]

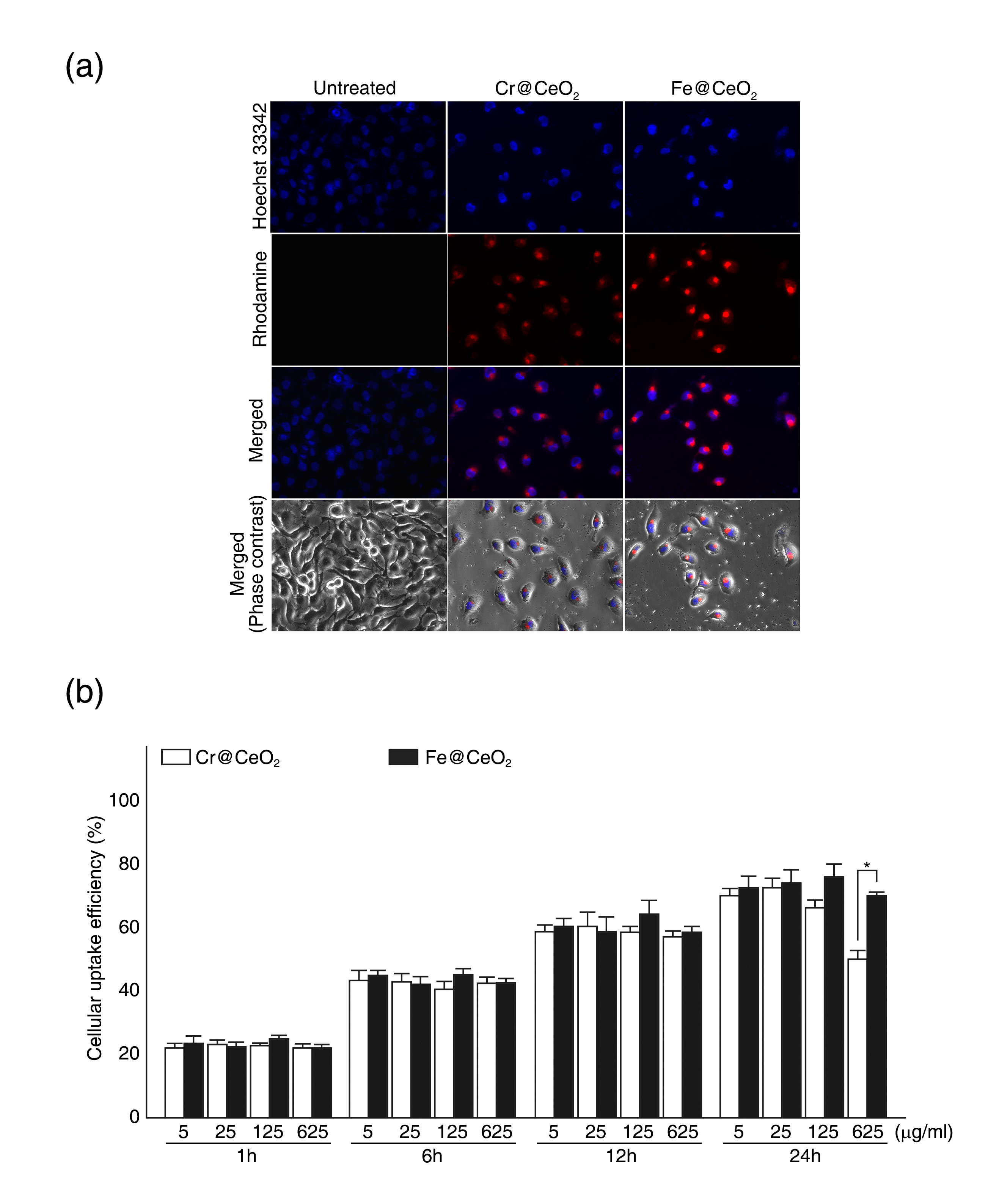

Supplement: Supplementary file 1 [file ijms-22-03087-s001.zip › Figure S2.jpg]

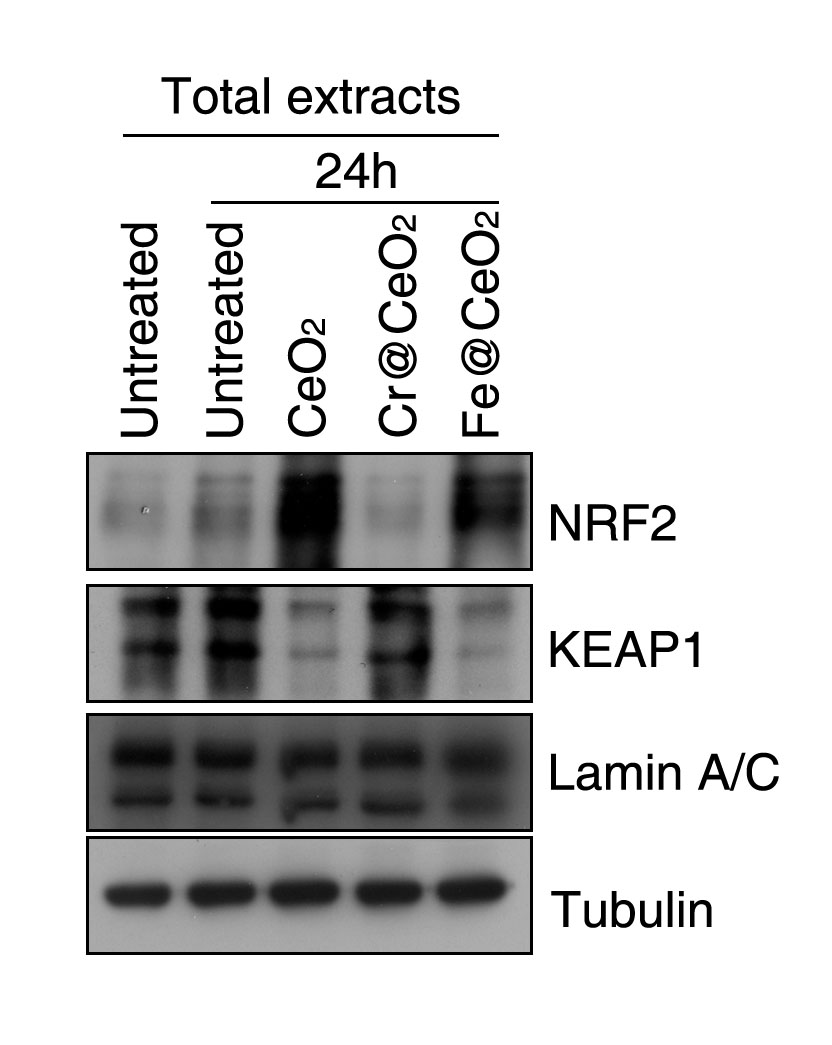

Supplement: Supplementary file 1 [file ijms-22-03087-s001.zip › Figure S3.jpg]
